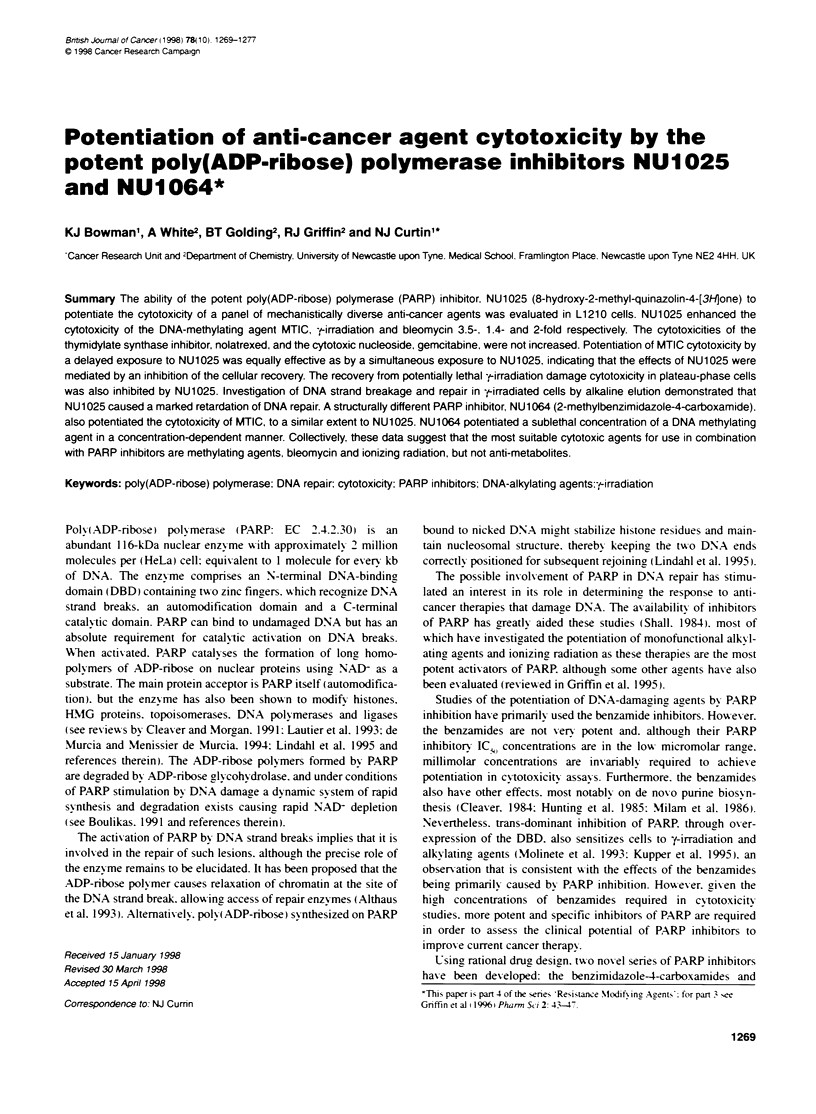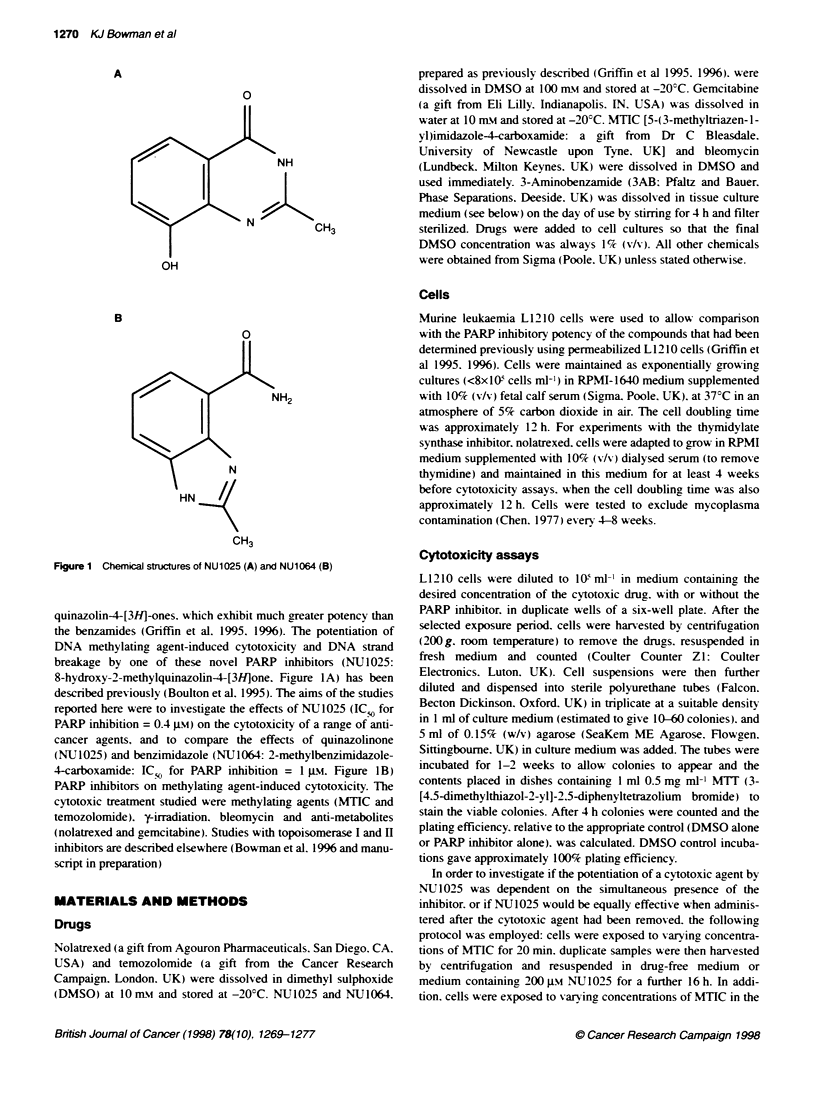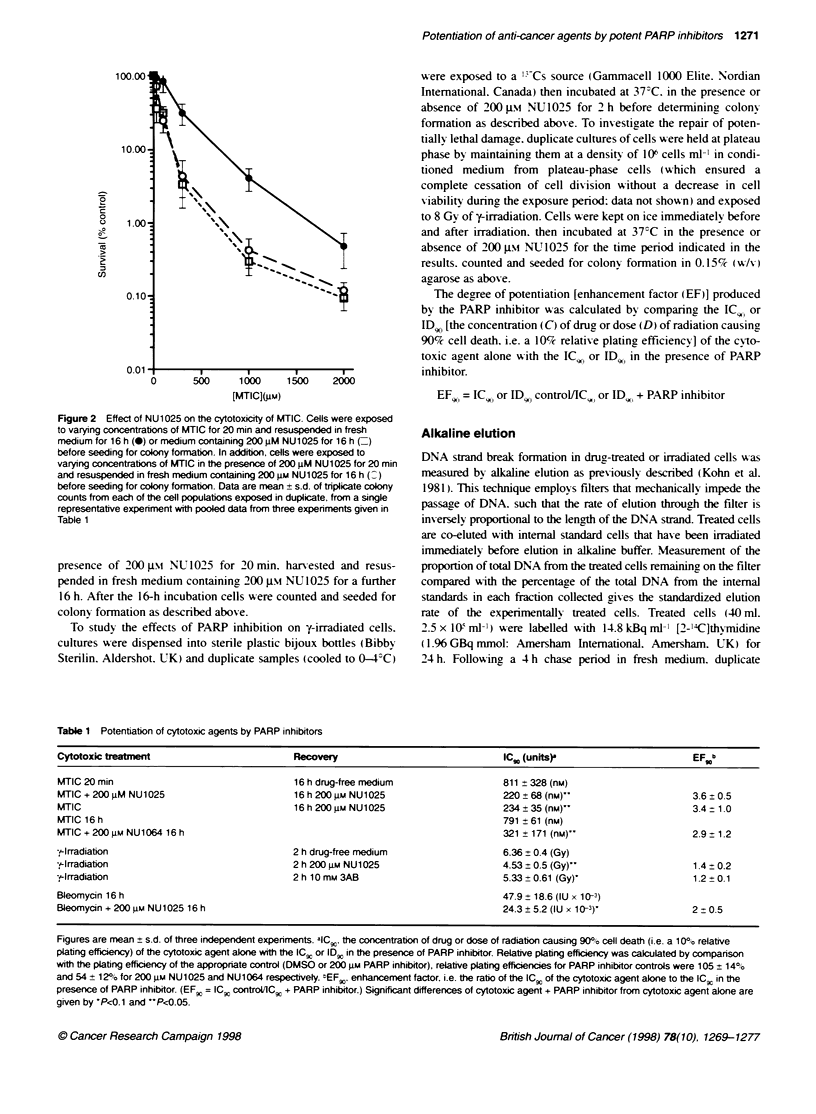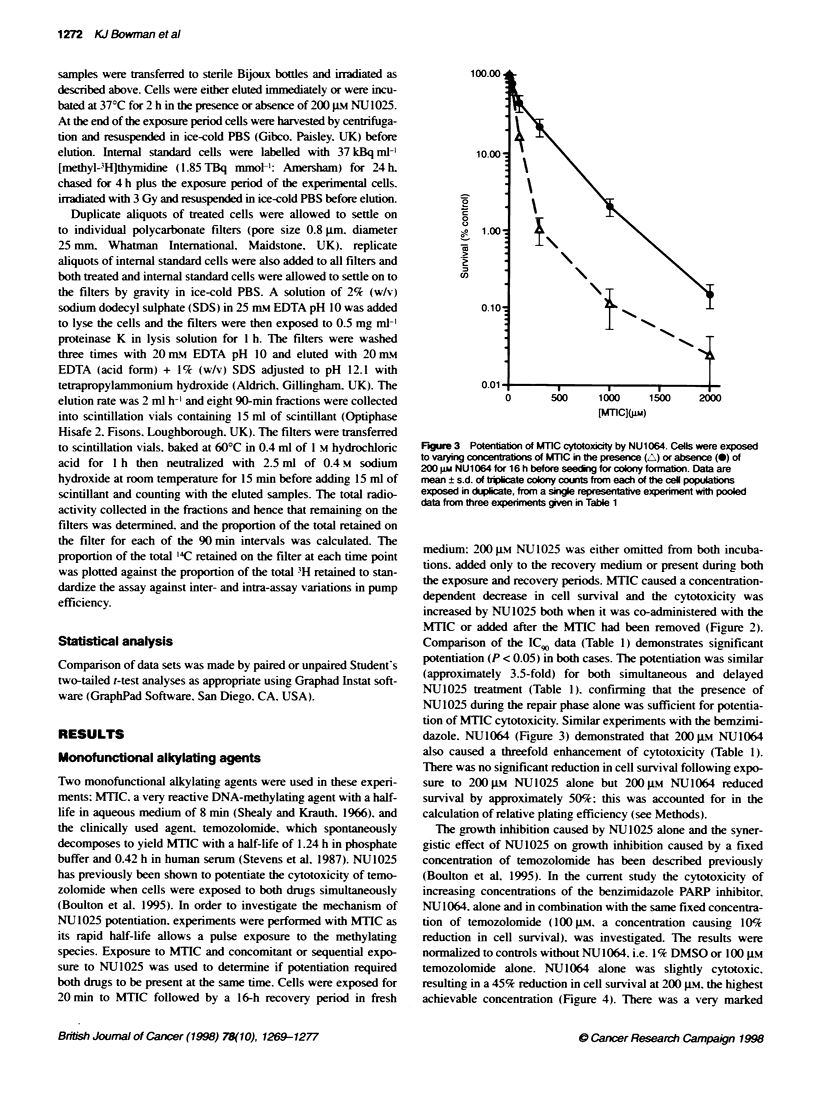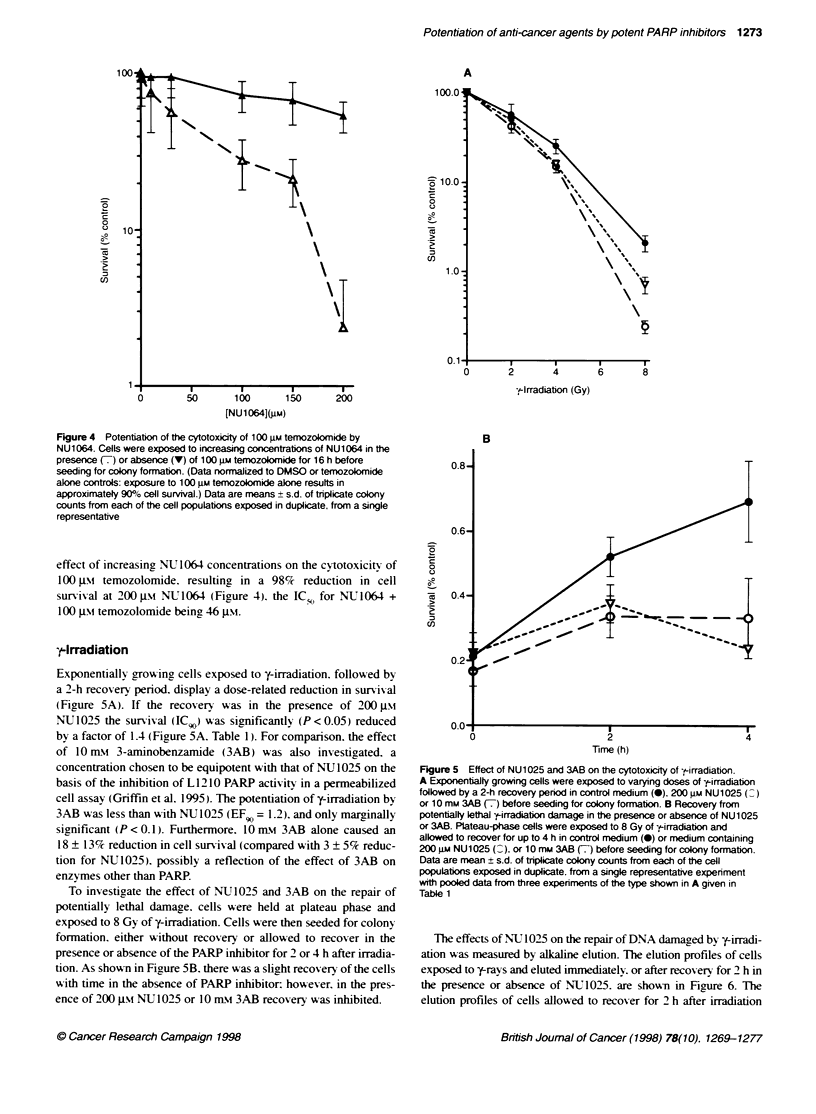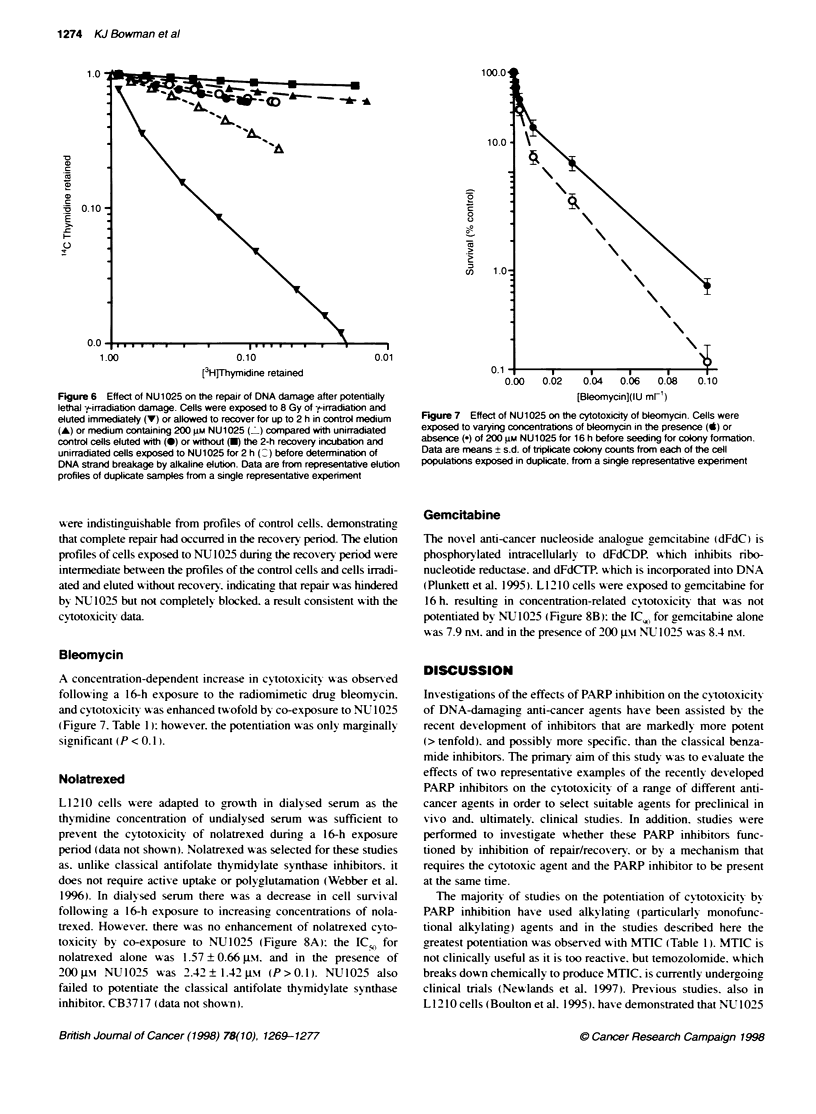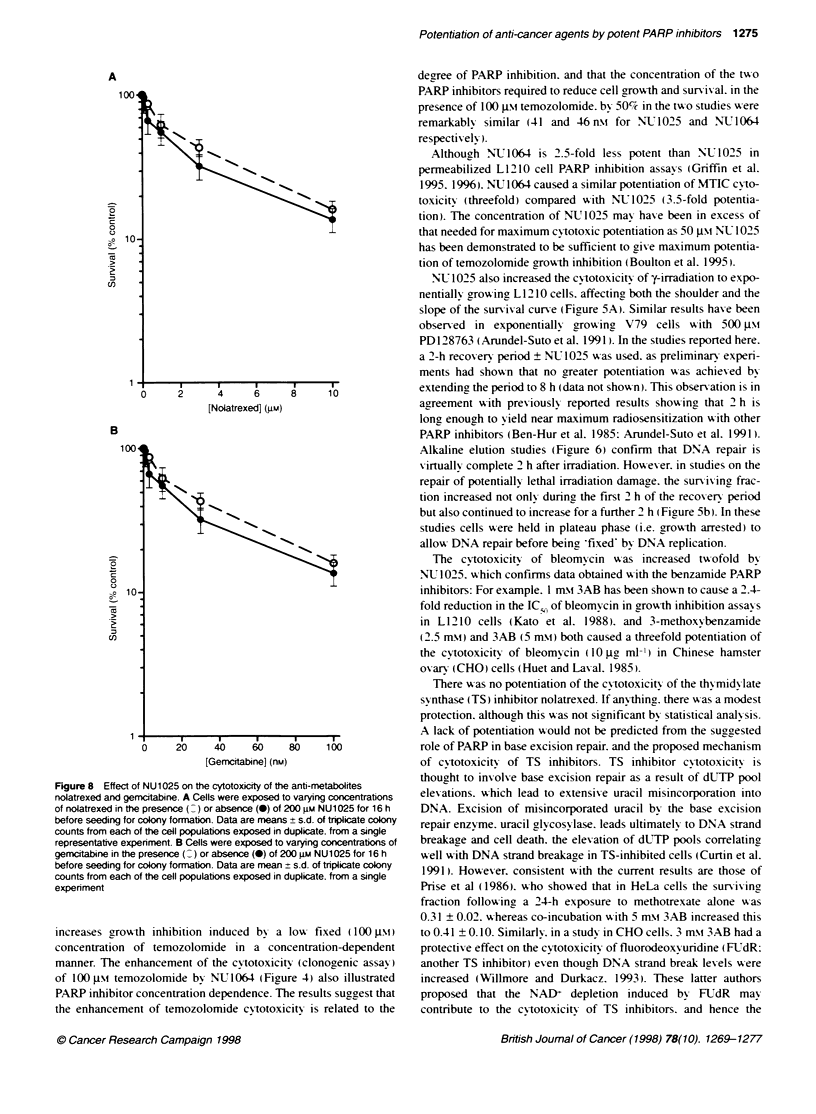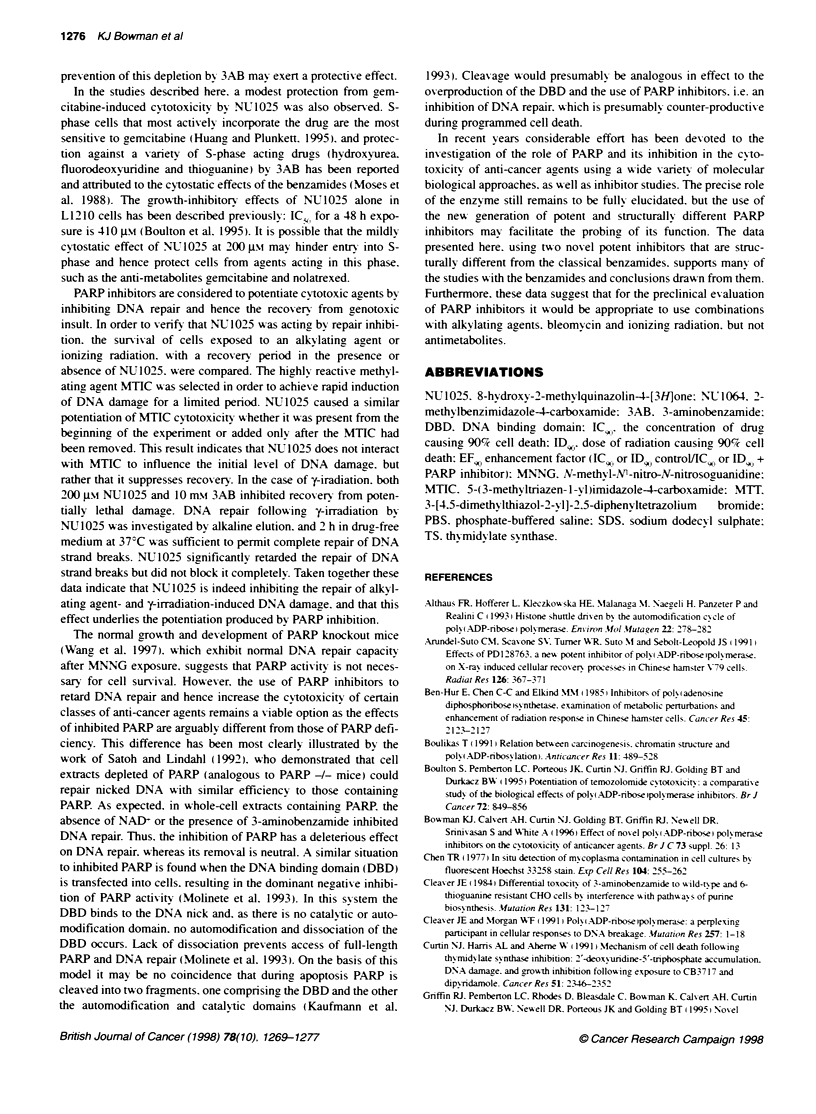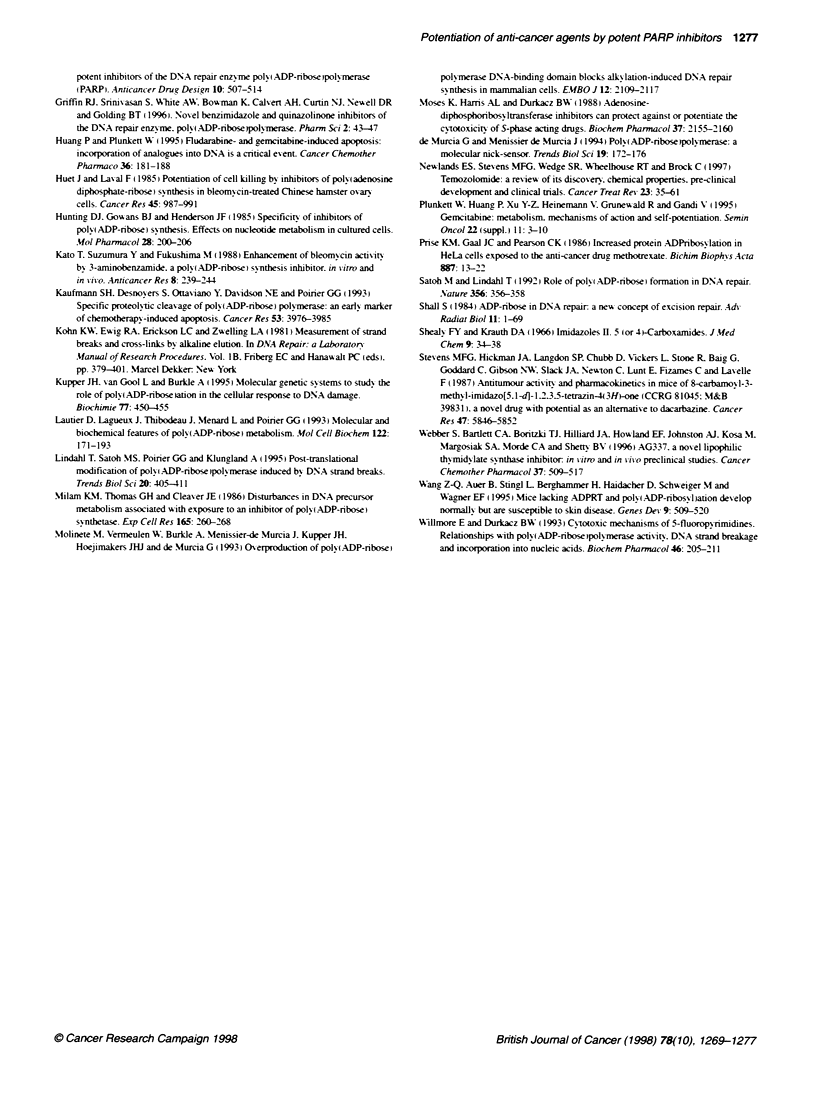# Potentiation of anti-cancer agent cytotoxicity by the potent poly(ADP-ribose) polymerase inhibitors NU1025 and NU1064.

**DOI:** 10.1038/bjc.1998.670

**Published:** 1998-11

**Authors:** K. J. Bowman, A. White, B. T. Golding, R. J. Griffin, N. J. Curtin

**Affiliations:** Cancer Research Unit, University of Newcastle upon Tyne, Medical School, UK.

## Abstract

The ability of the potent poly(ADP-ribose) polymerase (PARP) inhibitor, NU1025 (8-hydroxy-2-methyl-quinazolin-4-[3H]one) to potentiate the cytotoxicity of a panel of mechanistically diverse anti-cancer agents was evaluated in L1210 cells. NU1025 enhanced the cytotoxicity of the DNA-methylating agent MTIC, gamma-irradiation and bleomycin 3.5-, 1.4- and 2-fold respectively. The cytotoxicities of the thymidylate synthase inhibitor, nolatrexed, and the cytotoxic nucleoside, gemcitabine, were not increased. Potentiation of MTIC cytotoxicity by a delayed exposure to NU1025 was equally effective as by a simultaneous exposure to NU1025, indicating that the effects of NU1025 were mediated by an inhibition of the cellular recovery. The recovery from potentially lethal gamma-irradiation damage cytotoxicity in plateau-phase cells was also inhibited by NU1025. Investigation of DNA strand breakage and repair in gamma-irradiated cells by alkaline elution demonstrated that NU1025 caused a marked retardation of DNA repair. A structurally different PARP inhibitor, NU1064 (2-methylbenzimidazole-4-carboxamide), also potentiated the cytotoxicity of MTIC, to a similar extent to NU1025. NU1064 potentiated a sublethal concentration of a DNA methylating agent in a concentration-dependent manner. Collectively, these data suggest that the most suitable cytotoxic agents for use in combination with PARP inhibitors are methylating agents, bleomycin and ionizing radiation, but not anti-metabolites.


British Journal of Cancer (1998) 78(10), 1269–1277

© 1998 Cancer Research Campaign

### Potentiation of anti-cancer agent cytotoxicity by the potent poly(ADP-ribose) polymerase inhibitors NU1025 and NU1064*

**KJ Bowman[1], A White[2], BT Golding[2], RJ Griffin[2] and NJ Curtin[1*]**

[1]Cancer Research Unit and [2]Department of Chemistry, University of Newcastle upon Tyne, Medical School, Framlington Place, Newcastle upon Tyne NE2 4HH, UK

**Summary** The ability of the potent poly(ADP-ribose) polymerase (PARP) inhibitor, NU1025 (8-hydroxy-2-methyl-quinazolin-4-[*3H*]one) to potentiate the cytotoxicity of a panel of mechanistically diverse anti-cancer agents was evaluated in L1210 cells. NU1025 enhanced the cytotoxicity of the DNA-methylating agent MTIC, $\gamma$-irradiation and bleomycin 3.5-, 1.4- and 2-fold respectively. The cytotoxicities of the thymidylate synthase inhibitor, nolatrexed, and the cytotoxic nucleoside, gemcitabine, were not increased. Potentiation of MTIC cytotoxicity by a delayed exposure to NU1025 was equally effective as by a simultaneous exposure to NU1025, indicating that the effects of NU1025 were mediated by an inhibition of the cellular recovery. The recovery from potentially lethal $\gamma$-irradiation damage cytotoxicity in plateau-phase cells was also inhibited by NU1025. Investigation of DNA strand breakage and repair in $\gamma$-irradiated cells by alkaline elution demonstrated that NU1025 caused a marked retardation of DNA repair. A structurally different PARP inhibitor, NU1064 (2-methylbenzimidazole-4-carboxamide), also potentiated the cytotoxicity of MTIC, to a similar extent to NU1025. NU1064 potentiated a sublethal concentration of a DNA methylating agent in a concentration-dependent manner. Collectively, these data suggest that the most suitable cytotoxic agents for use in combination with PARP inhibitors are methylating agents, bleomycin and ionizing radiation, but not anti-metabolites.

**Keywords:** poly(ADP-ribose) polymerase; DNA repair; cytotoxicity; PARP inhibitors; DNA-alkylating agents; $\gamma$-irradiation

Poly(ADP-ribose) polymerase (PARP: EC 2.4.2.30) is an abundant 116-kDa nuclear enzyme with approximately 2 million molecules per (HeLa) cell; equivalent to 1 molecule for every kb of DNA. The enzyme comprises an N-terminal DNA-binding domain (DBD) containing two zinc fingers, which recognize DNA strand breaks, an automodification domain and a C-terminal catalytic domain. PARP can bind to undamaged DNA but has an absolute requirement for catalytic activation on DNA breaks. When activated, PARP catalyses the formation of long homopolymers of ADP-ribose on nuclear proteins using $NAD^+$ as a substrate. The main protein acceptor is PARP itself (automodification), but the enzyme has also been shown to modify histones, HMG proteins, topoisomerases, DNA polymerases and ligases (see reviews by Cleaver and Morgan, 1991; Lautier et al, 1993; de Murcia and Menissier de Murcia, 1994; Lindahl et al, 1995 and references therein). The ADP-ribose polymers formed by PARP are degraded by ADP-ribose glycohydrolase, and under conditions of PARP stimulation by DNA damage a dynamic system of rapid synthesis and degradation exists causing rapid $NAD^+$ depletion (see Boulikas, 1991 and references therein).

The activation of PARP by DNA strand breaks implies that it is involved in the repair of such lesions, although the precise role of the enzyme remains to be elucidated. It has been proposed that the ADP-ribose polymer causes relaxation of chromatin at the site of the DNA strand break, allowing access of repair enzymes (Althaus et al, 1993). Alternatively, poly(ADP-ribose) synthesized on PARP bound to nicked DNA might stabilize histone residues and maintain nucleosomal structure, thereby keeping the two DNA ends correctly positioned for subsequent rejoining (Lindahl et al, 1995).

The possible involvement of PARP in DNA repair has stimulated an interest in its role in determining the response to anti-cancer therapies that damage DNA. The availability of inhibitors of PARP has greatly aided these studies (Shall, 1984), most of which have investigated the potentiation of monofunctional alkylating agents and ionizing radiation as these therapies are the most potent activators of PARP, although some other agents have also been evaluated (reviewed in Griffin et al, 1995).

Studies of the potentiation of DNA-damaging agents by PARP inhibition have primarily used the benzamide inhibitors. However, the benzamides are not very potent and, although their PARP inhibitory $IC_{50}$ concentrations are in the low micromolar range, millimolar concentrations are invariably required to achieve potentiation in cytotoxicity assays. Furthermore, the benzamides also have other effects, most notably on de novo purine biosynthesis (Cleaver, 1984; Hunting et al, 1985; Milam et al, 1986). Nevertheless, trans-dominant inhibition of PARP, through overexpression of the DBD, also sensitizes cells to $\gamma$-irradiation and alkylating agents (Molinete et al, 1993; Kupper et al, 1995), an observation that is consistent with the effects of the benzamides being primarily caused by PARP inhibition. However, given the high concentrations of benzamides required in cytotoxicity studies, more potent and specific inhibitors of PARP are required in order to assess the clinical potential of PARP inhibitors to improve current cancer therapy.

Using rational drug design, two novel series of PARP inhibitors have been developed: the benzimidazole-4-carboxamides and

*Received 15 January 1998*
*Revised 30 March 1998*
*Accepted 15 April 1998*

*Correspondence to:* NJ Curtin

*This paper is part 4 of the series 'Resistance Modifying Agents': for part 3 see Griffin et al (1996) *Pharm Sci* 2: 43–47.

A

B

**Figure 1** Chemical structures of NU1025 (A) and NU1064 (B)

quinazolin-4-[3*H*]-ones, which exhibit much greater potency than the benzamides (Griffin et al. 1995, 1996). The potentiation of DNA methylating agent-induced cytotoxicity and DNA strand breakage by one of these novel PARP inhibitors (NU1025: 8-hydroxy-2-methylquinazolin-4-[3*H*]one, Figure 1A) has been described previously (Boulton et al. 1995). The aims of the studies reported here were to investigate the effects of NU1025 ($IC_{50}$ for PARP inhibition = 0.4 μM) on the cytotoxicity of a range of anticancer agents, and to compare the effects of quinazolinone (NU1025) and benzimidazole (NU1064: 2-methylbenzimidazole-4-carboxamide: $IC_{50}$ for PARP inhibition = 1 μM, Figure 1B) PARP inhibitors on methylating agent-induced cytotoxicity. The cytotoxic treatment studied were methylating agents (MTIC and temozolomide), γ-irradiation, bleomycin and anti-metabolites (nolatrexed and gemcitabine). Studies with topoisomerase I and II inhibitors are described elsewhere (Bowman et al. 1996 and manuscript in preparation)

### MATERIALS AND METHODS

### Drugs

Nolatrexed (a gift from Agouron Pharmaceuticals, San Diego, CA, USA) and temozolomide (a gift from the Cancer Research Campaign, London, UK) were dissolved in dimethyl sulphoxide (DMSO) at 10 mM and stored at −20°C. NU1025 and NU1064, prepared as previously described (Griffin et al 1995, 1996), were dissolved in DMSO at 100 mM and stored at −20°C. Gemcitabine (a gift from Eli Lilly, Indianapolis, IN, USA) was dissolved in water at 10 mM and stored at −20°C. MTIC [5-(3-methyltriazen-1-yl)imidazole-4-carboxamide: a gift from Dr C Bleasdale, University of Newcastle upon Tyne, UK] and bleomycin (Lundbeck, Milton Keynes, UK) were dissolved in DMSO and used immediately. 3-Aminobenzamide (3AB: Pfaltz and Bauer, Phase Separations, Deeside, UK) was dissolved in tissue culture medium (see below) on the day of use by stirring for 4 h and filter sterilized. Drugs were added to cell cultures so that the final DMSO concentration was always 1% (v/v). All other chemicals were obtained from Sigma (Poole, UK) unless stated otherwise.

### Cells

Murine leukaemia L1210 cells were used to allow comparison with the PARP inhibitory potency of the compounds that had been determined previously using permeabilized L1210 cells (Griffin et al 1995, 1996). Cells were maintained as exponentially growing cultures ($<8\times10^5$ cells $ml^{-1}$) in RPMI-1640 medium supplemented with 10% (v/v) fetal calf serum (Sigma, Poole, UK), at 37°C in an atmosphere of 5% carbon dioxide in air. The cell doubling time was approximately 12 h. For experiments with the thymidylate synthase inhibitor, nolatrexed, cells were adapted to grow in RPMI medium supplemented with 10% (v/v) dialysed serum (to remove thymidine) and maintained in this medium for at least 4 weeks before cytotoxicity assays, when the cell doubling time was also approximately 12 h. Cells were tested to exclude mycoplasma contamination (Chen, 1977) every 4–8 weeks.

### Cytotoxicity assays

L1210 cells were diluted to $10^5$ $ml^{-1}$ in medium containing the desired concentration of the cytotoxic drug, with or without the PARP inhibitor, in duplicate wells of a six-well plate. After the selected exposure period, cells were harvested by centrifugation (200 *g*, room temperature) to remove the drugs, resuspended in fresh medium and counted (Coulter Counter Z1: Coulter Electronics, Luton, UK). Cell suspensions were then further diluted and dispensed into sterile polyurethane tubes (Falcon, Becton Dickinson, Oxford, UK) in triplicate at a suitable density in 1 ml of culture medium (estimated to give 10–60 colonies), and 5 ml of 0.15% (w/v) agarose (SeaKem ME Agarose, Flowgen, Sittingbourne, UK) in culture medium was added. The tubes were incubated for 1–2 weeks to allow colonies to appear and the contents placed in dishes containing 1 ml 0.5 mg $ml^{-1}$ MTT (3-[4,5-dimethylthiazol-2-yl]-2,5-diphenyltetrazolium bromide) to stain the viable colonies. After 4 h colonies were counted and the plating efficiency, relative to the appropriate control (DMSO alone or PARP inhibitor alone), was calculated. DMSO control incubations gave approximately 100% plating efficiency.

In order to investigate if the potentiation of a cytotoxic agent by NU1025 was dependent on the simultaneous presence of the inhibitor, or if NU1025 would be equally effective when administered after the cytotoxic agent had been removed, the following protocol was employed: cells were exposed to varying concentrations of MTIC for 20 min, duplicate samples were then harvested by centrifugation and resuspended in drug-free medium or medium containing 200 μM NU1025 for a further 16 h. In addition, cells were exposed to varying concentrations of MTIC in the

© Cancer Research Campaign 1998

**Figure 2** Effect of NU1025 on the cytotoxicity of MTIC. Cells were exposed to varying concentrations of MTIC for 20 min and resuspended in fresh medium for 16 h (●) or medium containing 200 μM NU1025 for 16 h (□) before seeding for colony formation. In addition, cells were exposed to varying concentrations of MTIC in the presence of 200 μM NU1025 for 20 min and resuspended in fresh medium containing 200 μM NU1025 for 16 h (○) before seeding for colony formation. Data are mean ± s.d. of triplicate colony counts from each of the cell populations exposed in duplicate, from a single representative experiment with pooled data from three experiments given in Table 1

presence of 200 μM NU1025 for 20 min, harvested and resuspended in fresh medium containing 200 μM NU1025 for a further 16 h. After the 16-h incubation cells were counted and seeded for colony formation as described above.

To study the effects of PARP inhibition on γ-irradiated cells, cultures were dispensed into sterile plastic bijoux bottles (Bibby Sterilin, Aldershot, UK) and duplicate samples (cooled to 0–4°C) were exposed to a $^{137}Cs$ source (Gammacell 1000 Elite, Nordian International, Canada) then incubated at 37°C, in the presence or absence of 200 μM NU1025 for 2 h before determining colony formation as described above. To investigate the repair of potentially lethal damage, duplicate cultures of cells were held at plateau phase by maintaining them at a density of $10^6$ cells $ml^{-1}$ in conditioned medium from plateau-phase cells (which ensured a complete cessation of cell division without a decrease in cell viability during the exposure period; data not shown) and exposed to 8 Gy of γ-irradiation. Cells were kept on ice immediately before and after irradiation, then incubated at 37°C in the presence or absence of 200 μM NU1025 for the time period indicated in the results, counted and seeded for colony formation in 0.15% (w/v) agarose as above.

The degree of potentiation [enhancement factor (EF)] produced by the PARP inhibitor was calculated by comparing the $IC_{90}$ or $ID_{90}$ [the concentration (*C*) of drug or dose (*D*) of radiation causing 90% cell death, i.e. a 10% relative plating efficiency] of the cytotoxic agent alone with the $IC_{90}$ or $ID_{90}$ in the presence of PARP inhibitor.

$$EF_{90} = IC_{90} \text{ or } ID_{90} \text{ control}/IC_{90} \text{ or } ID_{90} + \text{PARP inhibitor}$$

### Alkaline elution

DNA strand break formation in drug-treated or irradiated cells was measured by alkaline elution as previously described (Kohn et al, 1981). This technique employs filters that mechanically impede the passage of DNA, such that the rate of elution through the filter is inversely proportional to the length of the DNA strand. Treated cells are co-eluted with internal standard cells that have been irradiated immediately before elution in alkaline buffer. Measurement of the proportion of total DNA from the treated cells remaining on the filter compared with the percentage of the total DNA from the internal standards in each fraction collected gives the standardized elution rate of the experimentally treated cells. Treated cells (40 ml, $2.5 \times 10^5$ $ml^{-1}$) were labelled with 14.8 kBq $ml^{-1}$ [2-$^{14}C$]thymidine (1.96 GBq mmol; Amersham International, Amersham, UK) for 24 h. Following a 4 h chase period in fresh medium, duplicate

**Table 1** Potentiation of cytotoxic agents by PARP inhibitors

| Cytotoxic treatment | Recovery | $IC_{90}$ (units)[a] | $EF_{90}$[b] |
|---|---|---|---|
| MTIC 20 min | 16 h drug-free medium | 811 ± 328 (nM) | |
| MTIC + 200 μM NU1025 | 16 h 200 μM NU1025 | 220 ± 68 (nM)** | 3.6 ± 0.5 |
| MTIC | 16 h 200 μM NU1025 | 234 ± 35 (nM)** | 3.4 ± 1.0 |
| MTIC 16 h | | 791 ± 61 (nM) | |
| MTIC + 200 μM NU1064 16 h | | 321 ± 171 (nM)** | 2.9 ± 1.2 |
| γ-Irradiation | 2 h drug-free medium | 6.36 ± 0.4 (Gy) | |
| γ-Irradiation | 2 h 200 μM NU1025 | 4.53 ± 0.5 (Gy)** | 1.4 ± 0.2 |
| γ-Irradiation | 2 h 10 mM 3AB | 5.33 ± 0.61 (Gy)* | 1.2 ± 0.1 |
| Bleomycin 16 h | | 47.9 ± 18.6 (IU × $10^{-3}$) | |
| Bleomycin + 200 μM NU1025 16 h | | 24.3 ± 5.2 (IU × $10^{-3}$)* | 2 ± 0.5 |

Figures are mean ± s.d. of three independent experiments. [a]$IC_{90}$, the concentration of drug or dose of radiation causing 90% cell death (i.e. a 10% relative plating efficiency) of the cytotoxic agent alone with the $IC_{90}$ or $ID_{90}$ in the presence of PARP inhibitor. Relative plating efficiency was calculated by comparison with the plating efficiency of the appropriate control (DMSO or 200 μM PARP inhibitor), relative plating efficiencies for PARP inhibitor controls were 105 ± 14% and 54 ± 12% for 200 μM NU1025 and NU1064 respectively. [b]$EF_{90}$, enhancement factor, i.e. the ratio of the $IC_{90}$ of the cytotoxic agent alone to the $IC_{90}$ in the presence of PARP inhibitor. ($EF_{90}$ = $IC_{90}$ control/$IC_{90}$ + PARP inhibitor.) Significant differences of cytotoxic agent + PARP inhibitor from cytotoxic agent alone are given by *$P$<0.1 and **$P$<0.05.

© Cancer Research Campaign 1998

samples were transferred to sterile Bijoux bottles and irradiated as described above. Cells were either eluted immediately or were incubated at 37°C for 2 h in the presence or absence of 200 μM NU1025. At the end of the exposure period cells were harvested by centrifugation and resuspended in ice-cold PBS (Gibco. Paisley. UK) before elution. Internal standard cells were labelled with 37 kBq ml$^{-1}$ [methyl-$^{3}$H]thymidine (1.85 TBq mmol$^{-1}$: Amersham) for 24 h. chased for 4 h plus the exposure period of the experimental cells. irradiated with 3 Gy and resuspended in ice-cold PBS before elution.

Duplicate aliquots of treated cells were allowed to settle on to individual polycarbonate filters (pore size 0.8 μm. diameter 25 mm. Whatman International. Maidstone. UK). replicate aliquots of internal standard cells were also added to all filters and both treated and internal standard cells were allowed to settle on to the filters by gravity in ice-cold PBS. A solution of 2% (w/v) sodium dodecyl sulphate (SDS) in 25 mM EDTA pH 10 was added to lyse the cells and the filters were then exposed to 0.5 mg ml$^{-1}$ proteinase K in lysis solution for 1 h. The filters were washed three times with 20 mM EDTA pH 10 and eluted with 20 mM EDTA (acid form) + 1% (w/v) SDS adjusted to pH 12.1 with tetrapropylammonium hydroxide (Aldrich. Gillingham. UK). The elution rate was 2 ml h$^{-1}$ and eight 90-min fractions were collected into scintillation vials containing 15 ml of scintillant (Optiphase Hisafe 2. Fisons. Loughborough. UK). The filters were transferred to scintillation vials. baked at 60°C in 0.4 ml of 1 M hydrochloric acid for 1 h then neutralized with 2.5 ml of 0.4 M sodium hydroxide at room temperature for 15 min before adding 15 ml of scintillant and counting with the eluted samples. The total radioactivity collected in the fractions and hence that remaining on the filters was determined. and the proportion of the total retained on the filter for each of the 90 min intervals was calculated. The proportion of the total $^{14}$C retained on the filter at each time point was plotted against the proportion of the total $^{3}$H retained to standardize the assay against inter- and intra-assay variations in pump efficiency.

### Statistical analysis

Comparison of data sets was made by paired or unpaired Student's two-tailed *t*-test analyses as appropriate using Graphad Instat software (GraphPad Software. San Diego. CA. USA).

### RESULTS

### Monofunctional alkylating agents

Two monofunctional alkylating agents were used in these experiments: MTIC. a very reactive DNA-methylating agent with a half-life in aqueous medium of 8 min (Shealy and Krauth. 1966). and the clinically used agent. temozolomide. which spontaneously decomposes to yield MTIC with a half-life of 1.24 h in phosphate buffer and 0.42 h in human serum (Stevens et al. 1987). NU1025 has previously been shown to potentiate the cytotoxicity of temozolomide when cells were exposed to both drugs simultaneously (Boulton et al. 1995). In order to investigate the mechanism of NU1025 potentiation. experiments were performed with MTIC as its rapid half-life allows a pulse exposure to the methylating species. Exposure to MTIC and concomitant or sequential exposure to NU1025 was used to determine if potentiation required both drugs to be present at the same time. Cells were exposed for 20 min to MTIC followed by a 16-h recovery period in fresh

**Figure 3** Potentiation of MTIC cytotoxicity by NU1064. Cells were exposed to varying concentrations of MTIC in the presence (△) or absence (●) of 200 μM NU1064 for 16 h before seeding for colony formation. Data are mean ± s.d. of triplicate colony counts from each of the cell populations exposed in duplicate, from a single representative experiment with pooled data from three experiments given in Table 1

medium: 200 μM NU1025 was either omitted from both incubations. added only to the recovery medium or present during both the exposure and recovery periods. MTIC caused a concentration-dependent decrease in cell survival and the cytotoxicity was increased by NU1025 both when it was co-administered with the MTIC or added after the MTIC had been removed (Figure 2). Comparison of the $IC_{90}$ data (Table 1) demonstrates significant potentiation ($P < 0.05$) in both cases. The potentiation was similar (approximately 3.5-fold) for both simultaneous and delayed NU1025 treatment (Table 1). confirming that the presence of NU1025 during the repair phase alone was sufficient for potentiation of MTIC cytotoxicity. Similar experiments with the bemzimidazole. NU1064 (Figure 3) demonstrated that 200 μM NU1064 also caused a threefold enhancement of cytotoxicity (Table 1). There was no significant reduction in cell survival following exposure to 200 μM NU1025 alone but 200 μM NU1064 reduced survival by approximately 50%: this was accounted for in the calculation of relative plating efficiency (see Methods).

The growth inhibition caused by NU1025 alone and the synergistic effect of NU1025 on growth inhibition caused by a fixed concentration of temozolomide has been described previously (Boulton et al. 1995). In the current study the cytotoxicity of increasing concentrations of the benzimidazole PARP inhibitor. NU1064. alone and in combination with the same fixed concentration of temozolomide (100 μM. a concentration causing 10% reduction in cell survival). was investigated. The results were normalized to controls without NU1064. i.e. 1% DMSO or 100 μM temozolomide alone. NU1064 alone was slightly cytotoxic. resulting in a 45% reduction in cell survival at 200 μM. the highest achievable concentration (Figure 4). There was a very marked

© Cancer Research Campaign 1998

**Figure 4** Potentiation of the cytotoxicity of 100 μM temozolomide by NU1064. Cells were exposed to increasing concentrations of NU1064 in the presence (△) or absence (▼) of 100 μM temozolomide for 16 h before seeding for colony formation. (Data normalized to DMSO or temozolomide alone controls: exposure to 100 μM temozolomide alone results in approximately 90% cell survival.) Data are means ± s.d. of triplicate colony counts from each of the cell populations exposed in duplicate, from a single representative effect of increasing NU1064 concentrations on the cytotoxicity of 100 μM temozolomide, resulting in a 98% reduction in cell survival at 200 μM NU1064 (Figure 4), the $IC_{50}$ for NU1064 + 100 μM temozolomide being 46 μM.

### γ-Irradiation

Exponentially growing cells exposed to γ-irradiation, followed by a 2-h recovery period, display a dose-related reduction in survival (Figure 5A). If the recovery was in the presence of 200 μM NU1025 the survival ($IC_{90}$) was significantly ($P < 0.05$) reduced by a factor of 1.4 (Figure 5A, Table 1). For comparison, the effect of 10 mM 3-aminobenzamide (3AB) was also investigated, a concentration chosen to be equipotent with that of NU1025 on the basis of the inhibition of L1210 PARP activity in a permeabilized cell assay (Griffin et al, 1995). The potentiation of γ-irradiation by 3AB was less than with NU1025 ($EF_{90}$ = 1.2), and only marginally significant ($P < 0.1$). Furthermore, 10 mM 3AB alone caused an 18 ± 13% reduction in cell survival (compared with 3 ± 5% reduction for NU1025), possibly a reflection of the effect of 3AB on enzymes other than PARP.

To investigate the effect of NU1025 and 3AB on the repair of potentially lethal damage, cells were held at plateau phase and exposed to 8 Gy of γ-irradiation. Cells were then seeded for colony formation, either without recovery or allowed to recover in the presence or absence of the PARP inhibitor for 2 or 4 h after irradiation. As shown in Figure 5B, there was a slight recovery of the cells with time in the absence of PARP inhibitor; however, in the presence of 200 μM NU1025 or 10 mM 3AB recovery was inhibited.

**Figure 5** Effect of NU1025 and 3AB on the cytotoxicity of γ-irradiation. **A** Exponentially growing cells were exposed to varying doses of γ-irradiation followed by a 2-h recovery period in control medium (●), 200 μM NU1025 (○) or 10 mM 3AB (▽) before seeding for colony formation. **B** Recovery from potentially lethal γ-irradiation damage in the presence or absence of NU1025 or 3AB. Plateau-phase cells were exposed to 8 Gy of γ-irradiation and allowed to recover for up to 4 h in control medium (●) or medium containing 200 μM NU1025 (○), or 10 mM 3AB (▽) before seeding for colony formation. Data are mean ± s.d. of triplicate colony counts from each of the cell populations exposed in duplicate, from a single representative experiment with pooled data from three experiments of the type shown in **A** given in Table 1

The effects of NU1025 on the repair of DNA damaged by γ-irradiation was measured by alkaline elution. The elution profiles of cells exposed to γ-rays and eluted immediately, or after recovery for 2 h in the presence or absence of NU1025, are shown in Figure 6. The elution profiles of cells allowed to recover for 2 h after irradiation

**Figure 6** Effect of NU1025 on the repair of DNA damage after potentially lethal γ-irradiation damage. Cells were exposed to 8 Gy of γ-irradiation and eluted immediately (▼) or allowed to recover for up to 2 h in control medium (▲) or medium containing 200 µM NU1025 (△) compared with unirradiated control cells eluted with (●) or without (■) the 2-h recovery incubation and unirradiated cells exposed to NU1025 for 2 h (○) before determination of DNA strand breakage by alkaline elution. Data are from representative elution profiles of duplicate samples from a single representative experiment

**Figure 7** Effect of NU1025 on the cytotoxicity of bleomycin. Cells were exposed to varying concentrations of bleomycin in the presence (●) or absence (○) of 200 µM NU1025 for 16 h before seeding for colony formation. Data are means ± s.d. of triplicate colony counts from each of the cell populations exposed in duplicate, from a single representative experiment were indistinguishable from profiles of control cells, demonstrating that complete repair had occurred in the recovery period. The elution profiles of cells exposed to NU1025 during the recovery period were intermediate between the profiles of the control cells and cells irradiated and eluted without recovery, indicating that repair was hindered by NU1025 but not completely blocked, a result consistent with the cytotoxicity data.

### Bleomycin

A concentration-dependent increase in cytotoxicity was observed following a 16-h exposure to the radiomimetic drug bleomycin, and cytotoxicity was enhanced twofold by co-exposure to NU1025 (Figure 7, Table 1); however, the potentiation was only marginally significant ($P < 0.1$).

### Nolatrexed

L1210 cells were adapted to growth in dialysed serum as the thymidine concentration of undialysed serum was sufficient to prevent the cytotoxicity of nolatrexed during a 16-h exposure period (data not shown). Nolatrexed was selected for these studies as, unlike classical antifolate thymidylate synthase inhibitors, it does not require active uptake or polyglutamation (Webber et al, 1996). In dialysed serum there was a decrease in cell survival following a 16-h exposure to increasing concentrations of nolatrexed. However, there was no enhancement of nolatrexed cytotoxicity by co-exposure to NU1025 (Figure 8A); the $IC_{50}$ for nolatrexed alone was 1.57 ± 0.66 µM, and in the presence of 200 µM NU1025 was 2.42 ± 1.42 µM ($P > 0.1$). NU1025 also failed to potentiate the classical antifolate thymidylate synthase inhibitor, CB3717 (data not shown).

### Gemcitabine

The novel anti-cancer nucleoside analogue gemcitabine (dFdC) is phosphorylated intracellularly to dFdCDP, which inhibits ribonucleotide reductase, and dFdCTP, which is incorporated into DNA (Plunkett et al, 1995). L1210 cells were exposed to gemcitabine for 16 h, resulting in concentration-related cytotoxicity that was not potentiated by NU1025 (Figure 8B); the $IC_{90}$ for gemcitabine alone was 7.9 nM, and in the presence of 200 µM NU1025 was 8.4 nM.

### DISCUSSION

Investigations of the effects of PARP inhibition on the cytotoxicity of DNA-damaging anti-cancer agents have been assisted by the recent development of inhibitors that are markedly more potent (> tenfold), and possibly more specific, than the classical benzamide inhibitors. The primary aim of this study was to evaluate the effects of two representative examples of the recently developed PARP inhibitors on the cytotoxicity of a range of different anti-cancer agents in order to select suitable agents for preclinical in vivo and, ultimately, clinical studies. In addition, studies were performed to investigate whether these PARP inhibitors functioned by inhibition of repair/recovery, or by a mechanism that requires the cytotoxic agent and the PARP inhibitor to be present at the same time.

The majority of studies on the potentiation of cytotoxicity by PARP inhibition have used alkylating (particularly monofunctional alkylating) agents and in the studies described here the greatest potentiation was observed with MTIC (Table 1). MTIC is not clinically useful as it is too reactive, but temozolomide, which breaks down chemically to produce MTIC, is currently undergoing clinical trials (Newlands et al, 1997). Previous studies, also in L1210 cells (Boulton et al, 1995), have demonstrated that NU1025

 © Cancer Research Campaign 1998

**Figure 8** Effect of NU1025 on the cytotoxicity of the anti-metabolites nolatrexed and gemcitabine. **A** Cells were exposed to varying concentrations of nolatrexed in the presence (○) or absence (●) of 200 μM NU1025 for 16 h before seeding for colony formation. Data are means ± s.d. of triplicate colony counts from each of the cell populations exposed in duplicate, from a single representative experiment. **B** Cells were exposed to varying concentrations of gemcitabine in the presence (○) or absence (●) of 200 μM NU1025 for 16 h before seeding for colony formation. Data are mean ± s.d. of triplicate colony counts from each of the cell populations exposed in duplicate, from a single experiment increases growth inhibition induced by a low fixed (100 μM) concentration of temozolomide in a concentration-dependent manner. The enhancement of the cytotoxicity (clonogenic assay) of 100 μM temozolomide by NU1064 (Figure 4) also illustrated PARP inhibitor concentration dependence. The results suggest that the enhancement of temozolomide cytotoxicity is related to the degree of PARP inhibition, and that the concentration of the two PARP inhibitors required to reduce cell growth and survival, in the presence of 100 μM temozolomide, by 50% in the two studies were remarkably similar (41 and 46 nM for NU1025 and NU1064 respectively).

Although NU1064 is 2.5-fold less potent than NU1025 in permeabilized L1210 cell PARP inhibition assays (Griffin et al, 1995, 1996), NU1064 caused a similar potentiation of MTIC cytotoxicity (threefold) compared with NU1025 (3.5-fold potentiation). The concentration of NU1025 may have been in excess of that needed for maximum cytotoxic potentiation as 50 μM NU1025 has been demonstrated to be sufficient to give maximum potentiation of temozolomide growth inhibition (Boulton et al, 1995).

NU1025 also increased the cytotoxicity of γ-irradiation to exponentially growing L1210 cells, affecting both the shoulder and the slope of the survival curve (Figure 5A). Similar results have been observed in exponentially growing V79 cells with 500 μM PD128763 (Arundel-Suto et al, 1991). In the studies reported here, a 2-h recovery period ± NU1025 was used, as preliminary experiments had shown that no greater potentiation was achieved by extending the period to 8 h (data not shown). This observation is in agreement with previously reported results showing that 2 h is long enough to yield near maximum radiosensitization with other PARP inhibitors (Ben-Hur et al, 1985; Arundel-Suto et al, 1991). Alkaline elution studies (Figure 6) confirm that DNA repair is virtually complete 2 h after irradiation. However, in studies on the repair of potentially lethal irradiation damage, the surviving fraction increased not only during the first 2 h of the recovery period but also continued to increase for a further 2 h (Figure 5b). In these studies cells were held in plateau phase (i.e. growth arrested) to allow DNA repair before being 'fixed' by DNA replication.

The cytotoxicity of bleomycin was increased twofold by NU1025, which confirms data obtained with the benzamide PARP inhibitors: For example, 1 mM 3AB has been shown to cause a 2.4-fold reduction in the $IC_{50}$ of bleomycin in growth inhibition assays in L1210 cells (Kato et al, 1988), and 3-methoxybenzamide (2.5 mM) and 3AB (5 mM) both caused a threefold potentiation of the cytotoxicity of bleomycin (10 μg $ml^{-1}$) in Chinese hamster ovary (CHO) cells (Huet and Laval, 1985).

There was no potentiation of the cytotoxicity of the thymidylate synthase (TS) inhibitor nolatrexed. If anything, there was a modest protection, although this was not significant by statistical analysis. A lack of potentiation would not be predicted from the suggested role of PARP in base excision repair, and the proposed mechanism of cytotoxicity of TS inhibitors. TS inhibitor cytotoxicity is thought to involve base excision repair as a result of dUTP pool elevations, which lead to extensive uracil misincorporation into DNA. Excision of misincorporated uracil by the base excision repair enzyme, uracil glycosylase, leads ultimately to DNA strand breakage and cell death, the elevation of dUTP pools correlating well with DNA strand breakage in TS-inhibited cells (Curtin et al, 1991). However, consistent with the current results are those of Prise et al (1986), who showed that in HeLa cells the surviving fraction following a 24-h exposure to methotrexate alone was 0.31 ± 0.02, whereas co-incubation with 5 mM 3AB increased this to 0.41 ± 0.10. Similarly, in a study in CHO cells, 3 mM 3AB had a protective effect on the cytotoxicity of fluorodeoxyuridine (FUdR; another TS inhibitor) even though DNA strand break levels were increased (Willmore and Durkacz, 1993). These latter authors proposed that the $NAD^+$ depletion induced by FUdR may contribute to the cytotoxicity of TS inhibitors, and hence the

© *Cancer Research Campaign 1998*

prevention of this depletion by 3AB may exert a protective effect.

In the studies described here, a modest protection from gemcitabine-induced cytotoxicity by NU1025 was also observed. S-phase cells that most actively incorporate the drug are the most sensitive to gemcitabine (Huang and Plunkett, 1995), and protection against a variety of S-phase acting drugs (hydroxyurea, fluorodeoxyuridine and thioguanine) by 3AB has been reported and attributed to the cytostatic effects of the benzamides (Moses et al, 1988). The growth-inhibitory effects of NU1025 alone in L1210 cells has been described previously: $IC_{50}$ for a 48 h exposure is 410 μM (Boulton et al, 1995). It is possible that the mildly cytostatic effect of NU1025 at 200 μM may hinder entry into S-phase and hence protect cells from agents acting in this phase, such as the anti-metabolites gemcitabine and nolatrexed.

PARP inhibitors are considered to potentiate cytotoxic agents by inhibiting DNA repair and hence the recovery from genotoxic insult. In order to verify that NU1025 was acting by repair inhibition, the survival of cells exposed to an alkylating agent or ionizing radiation, with a recovery period in the presence or absence of NU1025, were compared. The highly reactive methylating agent MTIC was selected in order to achieve rapid induction of DNA damage for a limited period. NU1025 caused a similar potentiation of MTIC cytotoxicity whether it was present from the beginning of the experiment or added only after the MTIC had been removed. This result indicates that NU1025 does not interact with MTIC to influence the initial level of DNA damage, but rather that it suppresses recovery. In the case of γ-iradiation, both 200 μM NU1025 and 10 mM 3AB inhibited recovery from potentially lethal damage. DNA repair following γ-irradiation by NU1025 was investigated by alkaline elution, and 2 h in drug-free medium at 37°C was sufficient to permit complete repair of DNA strand breaks. NU1025 significantly retarded the repair of DNA strand breaks but did not block it completely. Taken together these data indicate that NU1025 is indeed inhibiting the repair of alkylating agent- and γ-irradiation-induced DNA damage, and that this effect underlies the potentiation produced by PARP inhibition.

The normal growth and development of PARP knockout mice (Wang et al, 1997), which exhibit normal DNA repair capacity after MNNG exposure, suggests that PARP activity is not necessary for cell survival. However, the use of PARP inhibitors to retard DNA repair and hence increase the cytotoxicity of certain classes of anti-cancer agents remains a viable option as the effects of inhibited PARP are arguably different from those of PARP deficiency. This difference has been most clearly illustrated by the work of Satoh and Lindahl (1992), who demonstrated that cell extracts depleted of PARP (analogous to PARP –/– mice) could repair nicked DNA with similar efficiency to those containing PARP. As expected, in whole-cell extracts containing PARP, the absence of $NAD^+$ or the presence of 3-aminobenzamide inhibited DNA repair. Thus, the inhibition of PARP has a deleterious effect on DNA repair, whereas its removal is neutral. A similar situation to inhibited PARP is found when the DNA binding domain (DBD) is transfected into cells, resulting in the dominant negative inhibition of PARP activity (Molinete et al, 1993). In this system the DBD binds to the DNA nick and, as there is no catalytic or automodification domain, no automodification and dissociation of the DBD occurs. Lack of dissociation prevents access of full-length PARP and DNA repair (Molinete et al, 1993). On the basis of this model it may be no coincidence that during apoptosis PARP is cleaved into two fragments, one comprising the DBD and the other the automodification and catalytic domains (Kaufmann et al, 1993). Cleavage would presumably be analogous in effect to the overproduction of the DBD and the use of PARP inhibitors, i.e. an inhibition of DNA repair, which is presumably counter-productive during programmed cell death.

In recent years considerable effort has been devoted to the investigation of the role of PARP and its inhibition in the cytotoxicity of anti-cancer agents using a wide variety of molecular biological approaches, as well as inhibitor studies. The precise role of the enzyme still remains to be fully elucidated, but the use of the new generation of potent and structurally different PARP inhibitors may facilitate the probing of its function. The data presented here, using two novel potent inhibitors that are structurally different from the classical benzamides, supports many of the studies with the benzamides and conclusions drawn from them. Furthermore, these data suggest that for the preclinical evaluation of PARP inhibitors it would be appropriate to use combinations with alkylating agents, bleomycin and ionizing radiation, but not antimetabolites.

### ABBREVIATIONS

NU1025, 8-hydroxy-2-methylquinazolin-4-[3*H*]one; NU1064, 2-methylbenzimidazole-4-carboxamide; 3AB, 3-aminobenzamide; DBD, DNA binding domain; $IC_{90}$, the concentration of drug causing 90% cell death; $ID_{90}$, dose of radiation causing 90% cell death; $EF_{90}$ enhancement factor ($IC_{90}$ or $ID_{90}$ control/$IC_{90}$ or $ID_{90}$ + PARP inhibitor); MNNG, *N*-methyl-*N*′-nitro-*N*-nitrosoguanidine; MTIC, 5-(3-methyltriazen-1-yl)imidazole-4-carboxamide; MTT, 3-[4,5-dimethylthiazol-2-yl]-2,5-diphenyltetrazolium bromide; PBS, phosphate-buffered saline; SDS, sodium dodecyl sulphate; TS, thymidylate synthase.

### REFERENCES

Althaus FR, Hofferer L, Kleczkowska HE, Malanaga M, Naegeli H, Panzeter P and Realini C (1993) Histone shuttle driven by the automodification cycle of poly(ADP-ribose) polymerase. *Environ Mol Mutagen* **22**: 278–282

Arundel-Suto CM, Scavone SV, Turner WR, Suto M and Sebolt-Leopold JS (1991) Effects of PD128763, a new potent inhibitor of poly(ADP-ribose)polymerase, on X-ray induced cellular recovery processes in Chinese hamster V79 cells. *Radiat Res* **126**: 367–371

Ben-Hur E, Chen C-C and Elkind MM (1985) Inhibitors of poly(adenosine diphosphoribose)synthetase, examination of metabolic perturbations and enhancement of radiation response in Chinese hamster cells. *Cancer Res* **45**: 2123–2127

Boulikas T (1991) Relation between carcinogenesis, chromatin structure and poly(ADP-ribosylation). *Anticancer Res* **11**: 489–528

Boulton S, Pemberton LC, Porteous JK, Curtin NJ, Griffin RJ, Golding BT and Durkacz BW (1995) Potentiation of temozolomide cytotoxicity: a comparative study of the biological effects of poly(ADP-ribose)polymerase inhibitors. *Br J Cancer* **72**: 849–856

Bowman KJ, Calvert AH, Curtin NJ, Golding BT, Griffin RJ, Newell DR, Srinivasan S and White A (1996) Effect of novel poly(ADP-ribose) polymerase inhibitors on the cytotoxicity of anticancer agents. *Br J C* **73** suppl. 26: 13

Chen TR (1977) In situ detection of mycoplasma contamination in cell cultures by fluorescent Hoechst 33258 stain. *Exp Cell Res* **104**: 255–262

Cleaver JE (1984) Differential toxocity of 3-aminobenzamide to wild-type and 6-thioguanine resistant CHO cells by interference with pathways of purine biosynthesis. *Mutation Res* **131**: 123–127

Cleaver JE and Morgan WF (1991) Poly(ADP-ribose)polymerase: a perplexing participant in cellular responses to DNA breakage. *Mutation Res* **257**: 1–18

Curtin NJ, Harris AL and Aherne W (1991) Mechanism of cell death following thymidylate synthase inhibition: 2′-deoxyuridine-5′-triphosphate accumulation, DNA damage, and growth inhibition following exposure to CB3717 and dipyridamole. *Cancer Res* **51**: 2346–2352

Griffin RJ, Pemberton LC, Rhodes D, Bleasdale C, Bowman K, Calvert AH, Curtin NJ, Durkacz BW, Newell DR, Porteous JK and Golding BT (1995) Novel

*© Cancer Research Campaign 1998*

potent inhibitors of the DNA repair enzyme poly(ADP-ribose)polymerase (PARP). *Anticancer Drug Design* **10**: 507–514

Griffin RJ, Srinivasan S, White AW, Bowman K, Calvert AH, Curtin NJ, Newell DR and Golding BT (1996). Novel benzimidazole and quinazolinone inhibitors of the DNA repair enzyme, poly(ADP-ribose)polymerase. *Pharm Sci* **2**: 43–47

Huang P and Plunkett W (1995) Fludarabine- and gemcitabine-induced apoptosis: incorporation of analogues into DNA is a critical event. *Cancer Chemother Pharmaco* **36**: 181–188

Huet J and Laval F (1985) Potentiation of cell killing by inhibitors of poly(adenosine diphosphate-ribose) synthesis in bleomycin-treated Chinese hamster ovary cells. *Cancer Res* **45**: 987–991

Hunting DJ, Gowans BJ and Henderson JF (1985) Specificity of inhibitors of poly(ADP-ribose) synthesis. Effects on nucleotide metabolism in cultured cells. *Mol Pharmacol* **28**: 200–206

Kato T, Suzumura Y and Fukushima M (1988) Enhancement of bleomycin activity by 3-aminobenzamide, a poly(ADP-ribose) synthesis inhibitor, *in vitro* and *in vivo*. *Anticancer Res* **8**: 239–244

Kaufmann SH, Desnoyers S, Ottaviano Y, Davidson NE and Poirier GG (1993) Specific proteolytic cleavage of poly(ADP-ribose) polymerase: an early marker of chemotherapy-induced apoptosis. *Cancer Res* **53**: 3976–3985

Kohn KW, Ewig RA, Erickson LC and Zwelling LA (1981) Measurement of strand breaks and cross-links by alkaline elution. In *DNA Repair: a Laboratory Manual of Research Procedures*, Vol. 1B, Friberg EC and Hanawalt PC (eds), pp. 379–401. Marcel Dekker: New York

Kupper JH, van Gool L and Burkle A (1995) Molecular genetic systems to study the role of poly(ADP-ribose)ation in the cellular response to DNA damage. *Biochimie* **77**: 450–455

Lautier D, Lagueux J, Thibodeau J, Menard L and Poirier GG (1993) Molecular and biochemical features of poly(ADP-ribose) metabolism. *Mol Cell Biochem* **122**: 171–193

Lindahl T, Satoh MS, Poirier GG and Klungland A (1995) Post-translational modification of poly(ADP-ribose)polymerase induced by DNA strand breaks. *Trends Biol Sci* **20**: 405–411

Milam KM, Thomas GH and Cleaver JE (1986) Disturbances in DNA precursor metabolism associated with exposure to an inhibitor of poly(ADP-ribose) synthetase. *Exp Cell Res* **165**: 260–268

Molinete M, Vermeulen W, Burkle A, Menissier-de Murcia J, Kupper JH, Hoejimakers JHJ and de Murcia G (1993) Overproduction of poly(ADP-ribose) polymerase DNA-binding domain blocks alkylation-induced DNA repair synthesis in mammalian cells. *EMBO J* **12**: 2109–2117

Moses K, Harris AL and Durkacz BW (1988) Adenosine-diphosphoribosyltransferase inhibitors can protect against or potentiate the cytotoxicity of S-phase acting drugs. *Biochem Pharmacol* **37**: 2155–2160

de Murcia G and Menissier de Murcia J (1994) Poly(ADP-ribose)polymerase: a molecular nick-sensor. *Trends Biol Sci* **19**: 172–176

Newlands ES, Stevens MFG, Wedge SR, Wheelhouse RT and Brock C (1997) Temozolomide: a review of its discovery, chemical properties, pre-clinical development and clinical trials. *Cancer Treat Rev* **23**: 35–61

Plunkett W, Huang P, Xu Y-Z, Heinemann V, Grunewald R and Gandi V (1995) Gemcitabine: metabolism, mechanisms of action and self-potentiation. *Semin Oncol* **22** (suppl.) 11: 3–10

Prise KM, Gaal JC and Pearson CK (1986) Increased protein ADPribosylation in HeLa cells exposed to the anti-cancer drug methotrexate. *Bichim Biophys Acta* **887**: 13–22

Satoh M and Lindahl T (1992) Role of poly(ADP-ribose) formation in DNA repair. *Nature* **356**: 356–358

Shall S (1984) ADP-ribose in DNA repair: a new concept of excision repair. *Adv Radiat Biol* **11**: 1–69

Shealy FY and Krauth DA (1966) Imidazoles II. 5 (or 4)-Carboxamides. *J Med Chem* **9**: 34–38

Stevens MFG, Hickman JA, Langdon SP, Chubb D, Vickers L, Stone R, Baig G, Goddard C, Gibson NW, Slack JA, Newton C, Lunt E, Fizames C and Lavelle F (1987) Antitumour activity and pharmacokinetics in mice of 8-carbamoyl-3-methyl-imidazo[5,1-*d*]-1,2,3,5-tetrazin-4(3*H*)-one (CCRG 81045; M&B 39831), a novel drug with potential as an alternative to dacarbazine. *Cancer Res* **47**: 5846–5852

Webber S, Bartlett CA, Boritzki TJ, Hilliard JA, Howland EF, Johnston AJ, Kosa M, Margosiak SA, Morde CA and Shetty BV (1996) AG337, a novel lipophilic thymidylate synthase inhibitor: *in vitro* and *in vivo* preclinical studies. *Cancer Chemother Pharmacol* **37**: 509–517

Wang Z-Q, Auer B, Stingl L, Berghammer H, Haidacher D, Schweiger M and Wagner EF (1995) Mice lacking ADPRT and poly(ADP-ribosyl)ation develop normally but are susceptible to skin disease. *Genes Dev* **9**: 509–520

Willmore E and Durkacz BW (1993) Cytotoxic mechanisms of 5-fluoropyrimidines. Relationships with poly(ADP-ribose)polymerase activity, DNA strand breakage and incorporation into nucleic acids. *Biochem Pharmacol* **46**: 205–211

© Cancer Research Campaign 1998